# Supplementary material for: Early stopping in clinical PET studies: How to reduce expense and exposure
Source: J Cereb Blood Flow Metab. 2021 May 21;41(11):2805–19. doi: 10.1177/0271678X211017796 (PMC8545054; doi:10.1177/0271678X211017796)
Supplement: sj-pdf-1-jcb-10.1177_0271678X211017796 - Supplemental material for Early stopping in clinical PET studies: How to reduce expense and exposure [file sj-pdf-1-jcb-10.1177_0271678X211017796.pdf]

Supplementary information - Early stopping in clinical PET studies: how to reduce expense and exposure

The Bayes Factor

The Bayes Factor is also known as the "predictive updating factor" of Bayes Theorem. It is what updates the prior odds of two competing hypotheses into posterior odds after seeing the observed data, and hence describes the relative "predictive adequacy" of the hypotheses. Assuming that H_0 and H_1 have equal prior probability ($P(H_1) = P(H_0) = 0.5$), then a BF of e.g., 3 means that after conducting the study, H_1 is 3 times more likely than H_0, according to Bayes theorem:

$$\frac{P(H1|D)}{P(H0|D)} = BF * \frac{P(H1)}{P(H0)}$$

This corresponds to a posterior probability of 75% for H_1 and 25% for H_0. Although posterior odds are the natural extension of the BF, researchers tend to only report the BF as a stand-alone metric for assessing different hypotheses, due to the inherent subjective nature of specifying prior odds.

Functional form of the Cauchy distribution

The functional form of the Cauchy density function can be expressed as:

$$f(x; x_0, r) = \frac{1}{\pi r\left(1+\left(\frac{x-x_0}{r}\right)^2\right)},$$

where x_0 is the location parameter, specifying the location of the peak of the distribution, and r is the scale parameter which specifies the half-width at half-maximum.

Jeffrey's Bayesian t-test

The "default" BF t-test (using a zero-centered Cauchy to describe the prior over the parameter of interest under the alternative-hypothesis) is also known as Jeffreys's Bayesian t-test and can be calculated using the conventional *t*-statistic *(t):*

$$BF(n,t) = \frac{r\int_0^{\infty}(1+ng)^{-\frac{1}{2}}\left(1+\frac{t^2}{v(1+ng)}\right)^{-\frac{v+1}{2}}(2\pi)^{-\frac{1}{2}}g^{-\frac{3}{2}}e^{-\frac{r^2}{2g}}dg}{\left(1+\frac{t^2}{v}\right)^{-\frac{v+1}{2}}},$$

where *r* is the Cauchy the width parameter, *n* is the number of observations, *v* is *n*-1 (i.e., the degrees of freedom).

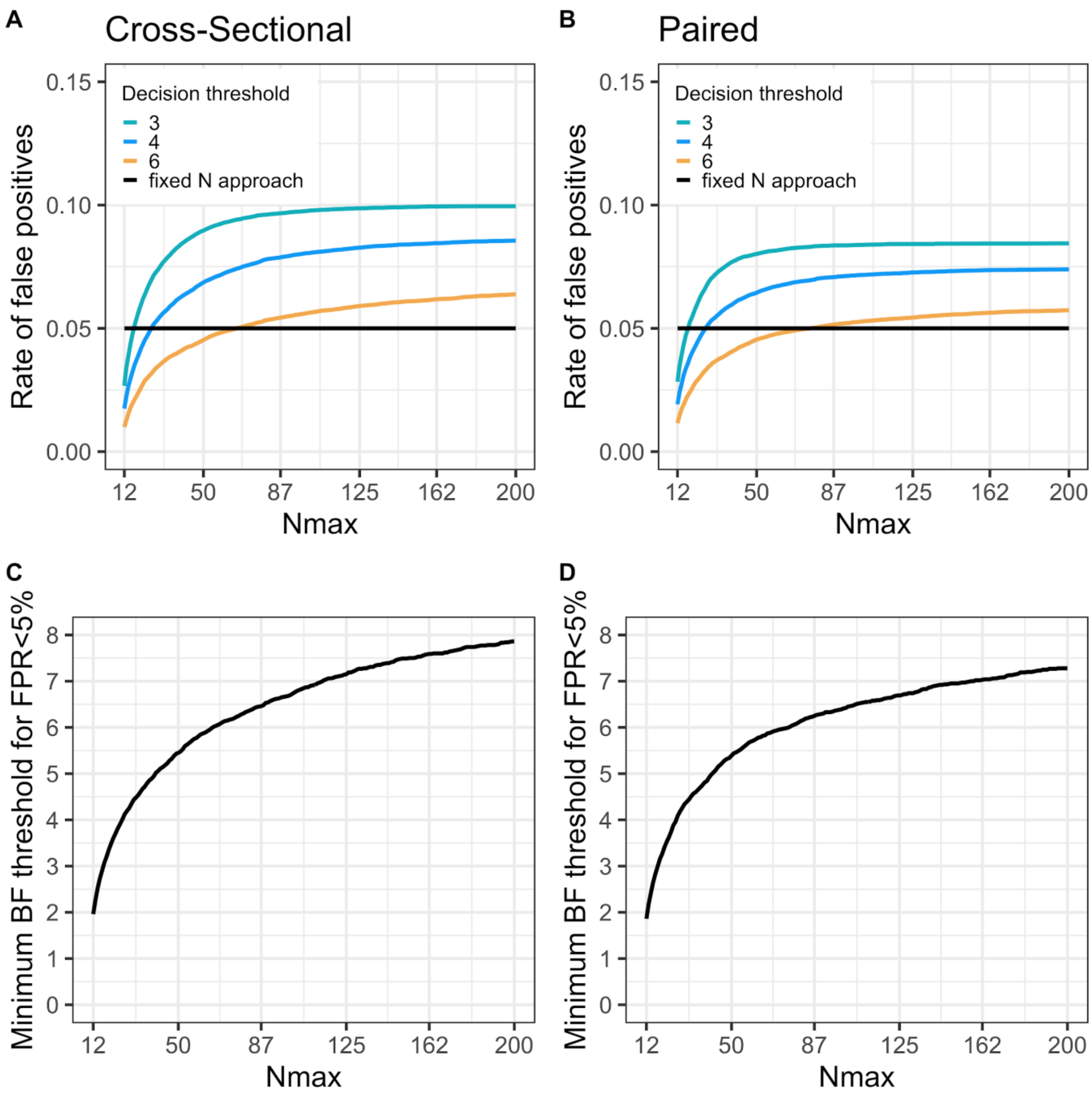

Figure S1. A and B) The rate of false positive stopping decisions increases but reaches an asymptote as the maximal number of subjects (Nmax) becomes higher. Three different BF decision thresholds are shown. C and D) The BF decision threshold can be adjusted to achieve a desired rate of false positives (here 5%) for different Nmax. For all figures: samples are drawn from two populations with the same mean value; testing starts at N =12/group; and BF is checked after every additional comparison pair (1 set of patient-control scans or pre-post scans). Here stop decisions for H0 are allowed and the tests are one-sided.

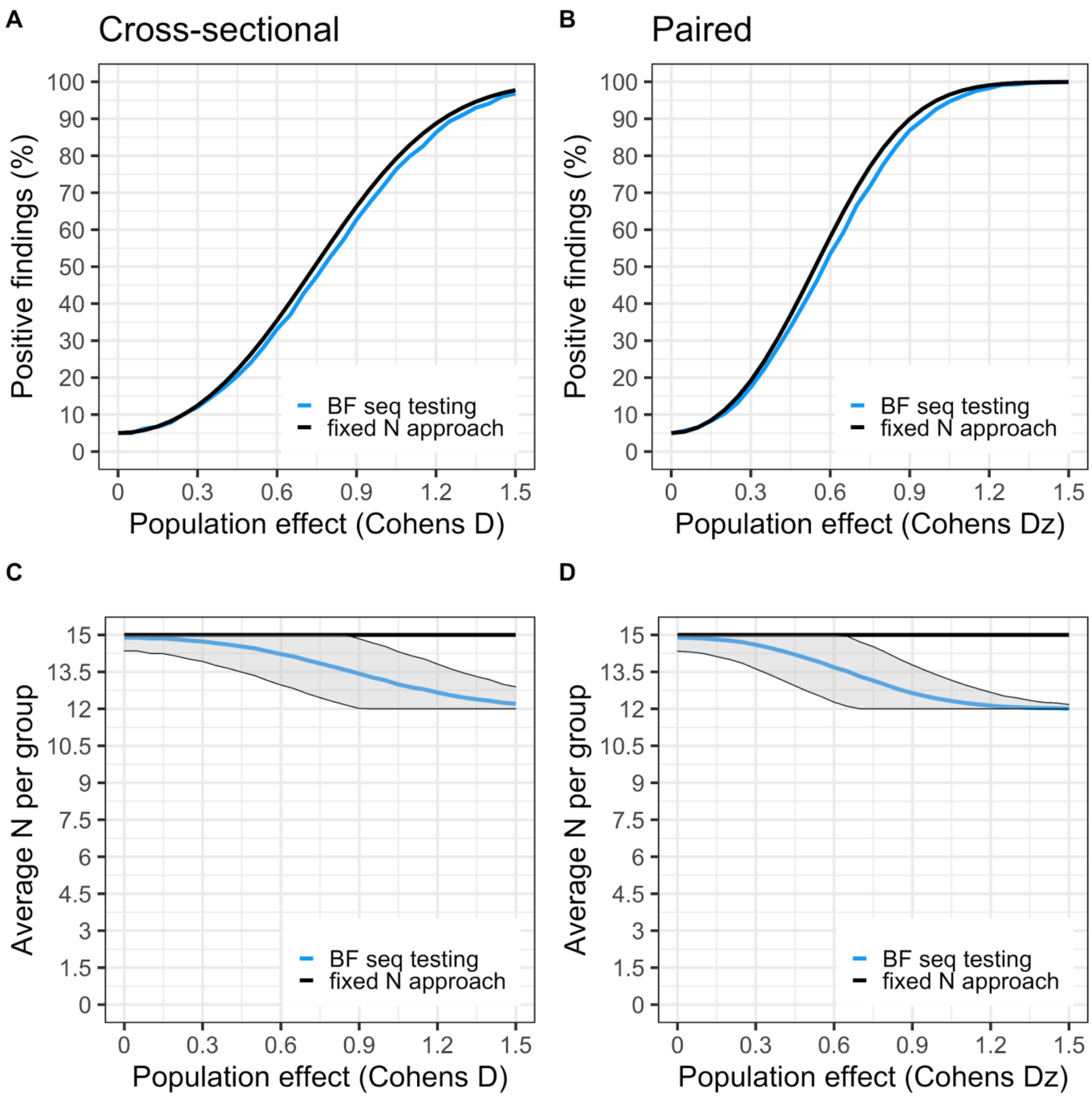

Figure S2. Settings for the simulation: H1 is described by a two-sided Cauchy (0,0.707), (Nstart = 12, Nmax = 15, BF threshold = 2.4 and 2.5 for cross-sectional and paired respectively). Panel A and B shows true positive (or "power") curves for BF sequential testing (blue) and a fixed N approach (black). The curves denote the rate of true positive findings at different population effects. For the fixed N approach, only one test is performed at N=15 per group. For the sequential testing, 8 subjects/group are first collected, then BF is checked after each added comparison pair until 15 subjects/group is reached, using a stopping threshold of 4. Panel C and D shows the average number of subjects needed to reach a stopping decision at different population effects. Fixed N is the black line (fixed at N = 15/group); BF sequential testing is the blue line with shaded area denoting ± 1 SD.

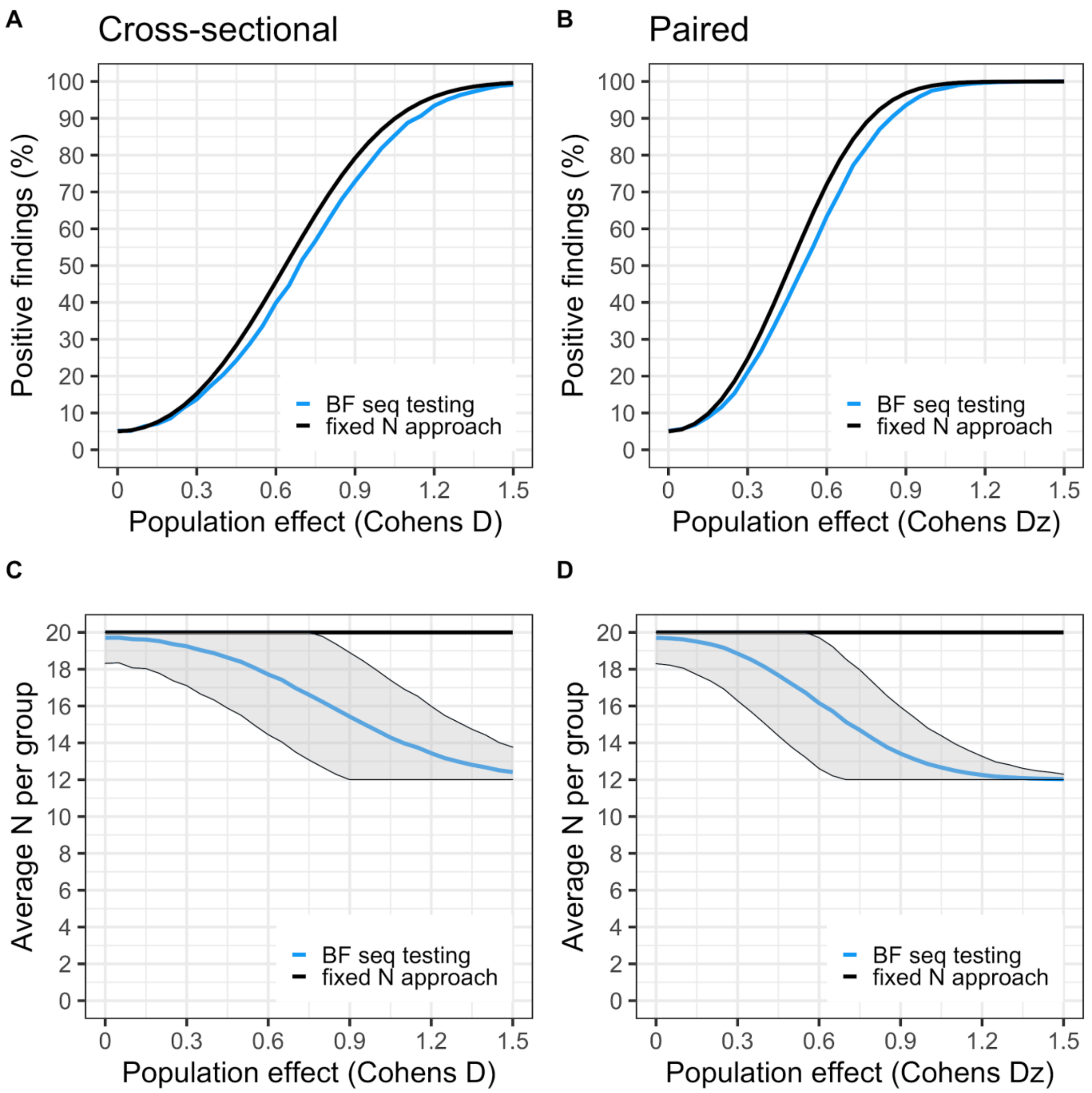

Figure S3. Settings for the simulation: H1 is described by a two-sided Cauchy (0,0.707), (Nstart = 12, Nmax = 20, BF threshold = 3.1 and 3.3 for cross-sectional and paired respectively). Panel A and B shows true positive (or "power") curves for BF sequential testing (blue) and a fixed N approach (black). The curves denote the rate of true positive findings at different population effects. For the fixed N approach, only one test is performed at N=20 per group. For the sequential testing, 12 subjects/group are first collected, then BF is checked after each added comparison pair until 20 subjects/group is reached, using a stopping threshold of 4. Panel C and D shows the average number of subjects needed to reach a stopping decision at different population effects. Fixed N is the black line (fixed at N = 20/group); BF sequential testing is the blue line with shaded area denoting ± 1 SD.

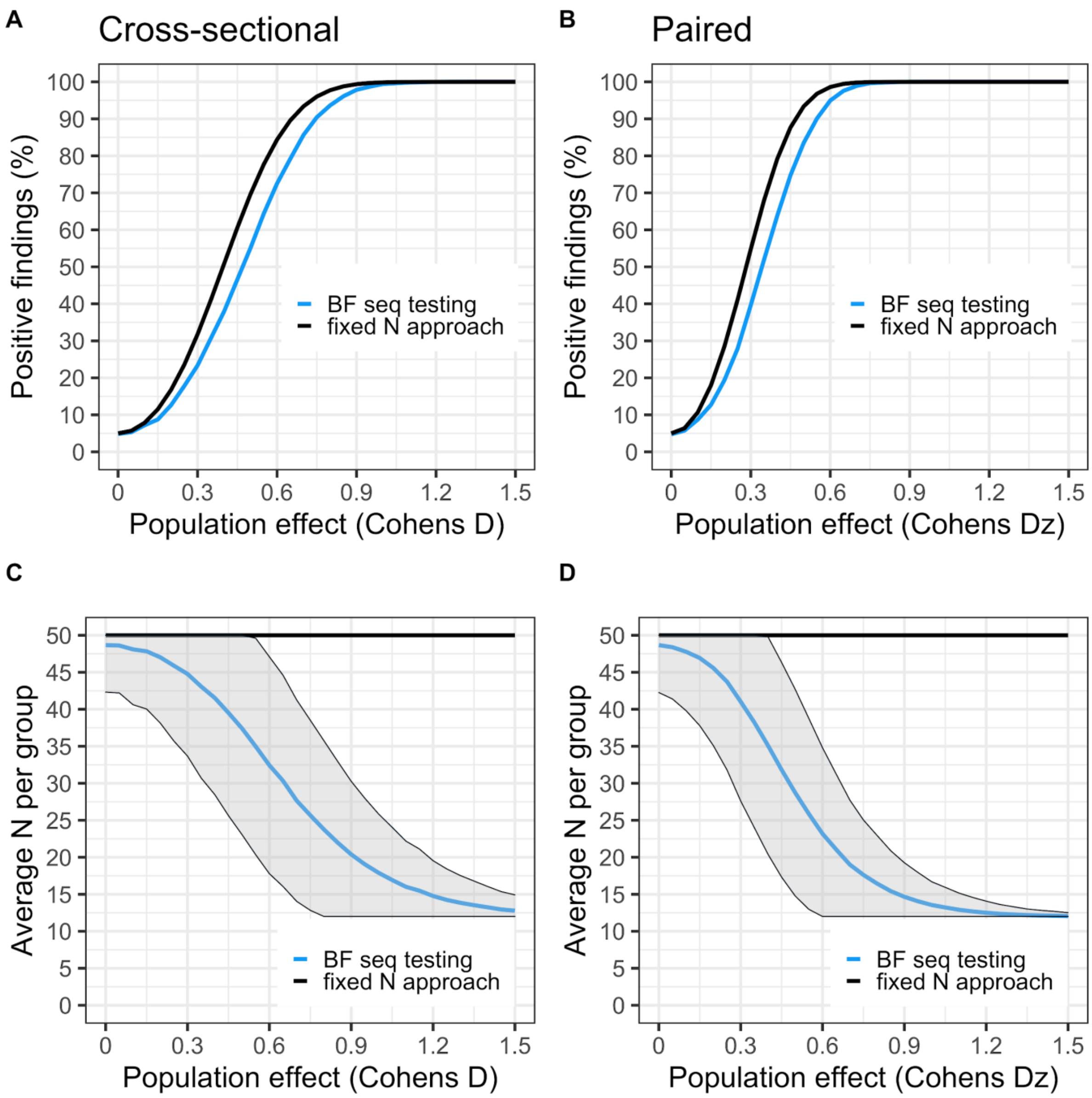

Figure S4. Settings for the simulation: H1 is described by a two-sided Cauchy (0,0.707), (Nstart = 12, Nmax = 50, BF threshold = 5.2 and 5.4 for cross-sectional and paired respectively). Panel A and B shows true positive (or "power") curves for BF sequential testing (blue) and a fixed N approach (black). The curves denote the rate of true positive findings at different population effects. For the fixed N approach, only one test is performed at N=50 per group. For the sequential testing, 12 subjects/group are first collected, then BF is checked after each added comparison pair until 50 subjects/group is reached, using a stopping threshold of 4. Panel C and D shows the average number of subjects needed to reach a stopping decision at different population effects. Fixed N is the black line (fixed at N = 50/group); BF sequential testing is the blue line with shaded area denoting ± 1 SD.

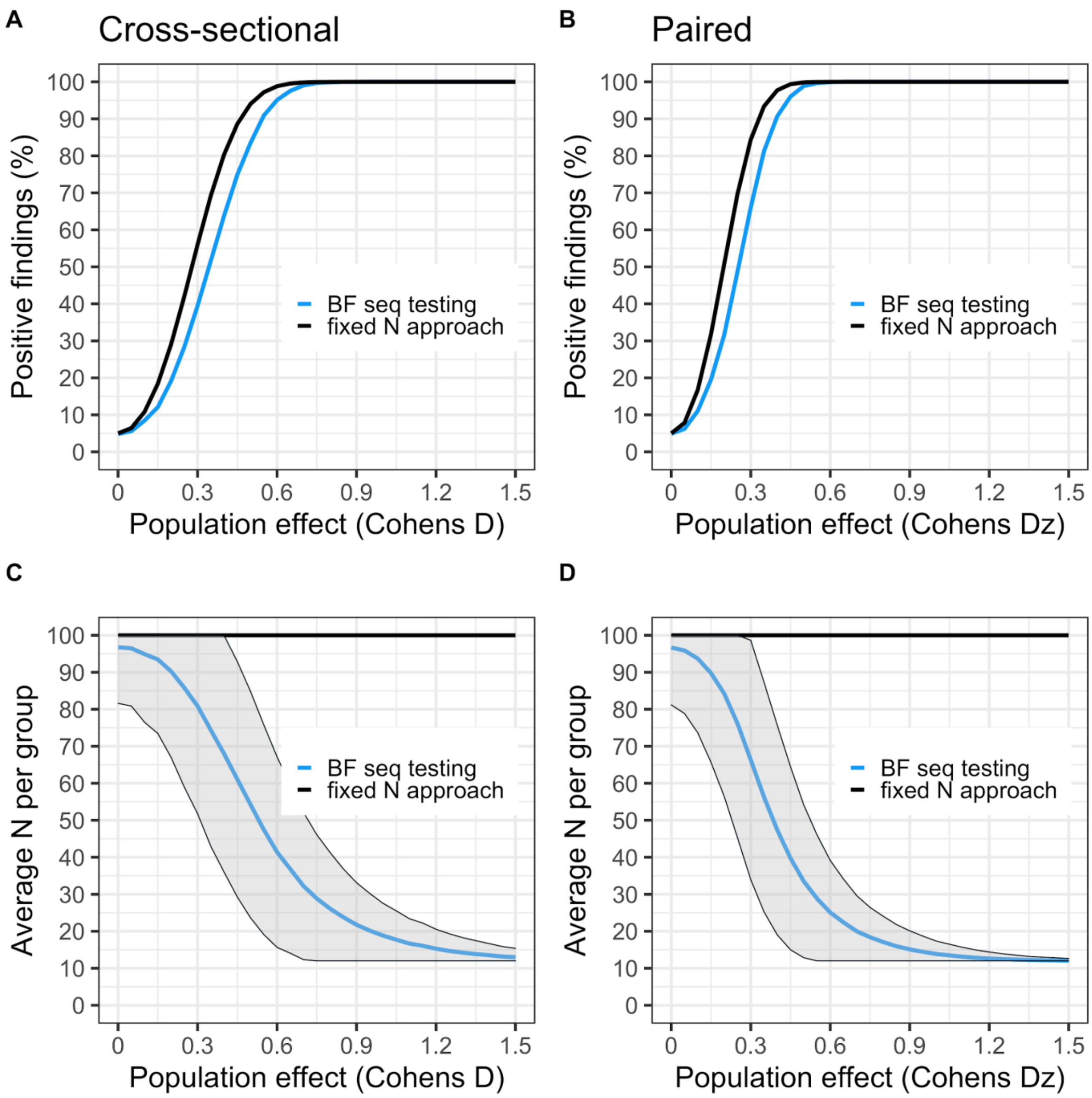

Figure S5. Settings for the simulation: H1 is described by a two-sided Cauchy(0,0.707), (Nstart = 12, Nmax = 100, BF threshold = 6.6 and 6.7 for cross-sectional and paired respectively). Panel A and B shows true positive (or "power") curves for BF sequential testing (blue) and fixed N approach (black). The curves denote the rate of true positive findings at different population effects. For the fixed N approach, only one test is performed at N=100 per group. For the sequential testing, 12 subjects/group are first collected, then BF is checked after each added comparison pair until 100 subjects/group is reached, using a stopping threshold of 4. Panel C and D shows the average number of subjects needed to reach a stopping decision at different population effects. Fixed N is the black line (fixed at N = 100/group); BF sequential testing is the blue line with shaded area denoting ± 1 SD.

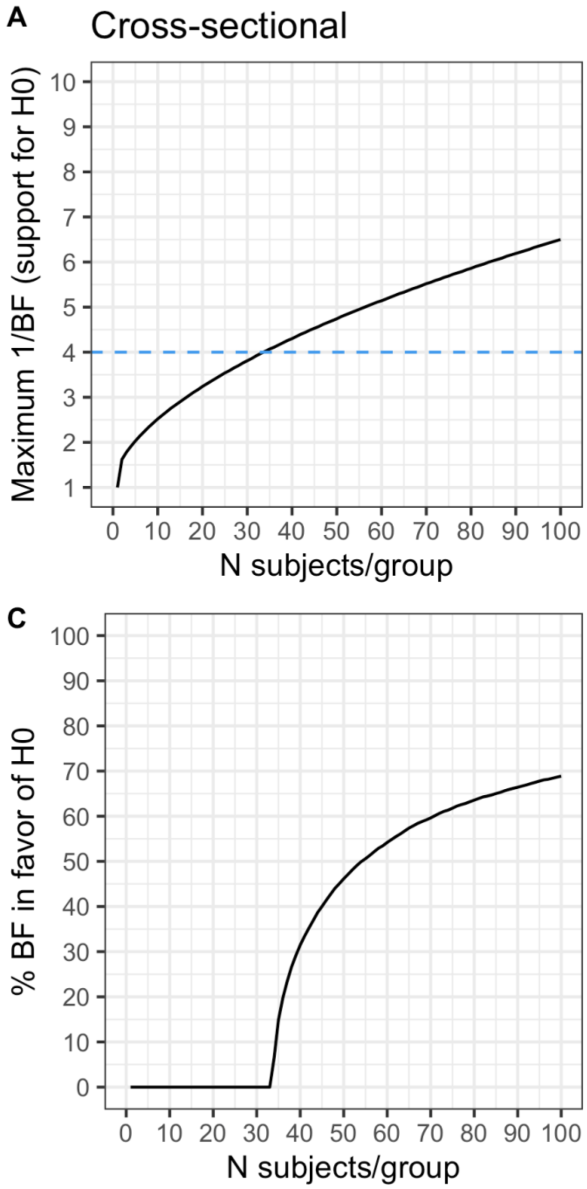

Figure S6. Maximum possible support (1/BF) in favor of H0 compared to H1 when using a two-tailed BF t-test. A-D) When using the settings described in the main article (H1 is specified as a Cauchy(0,0.707), Nstart = 12, threshold = 4) but a two-tailed test instead of a one-tailed test, it is not possible to obtained evidence in favor of H0 at smaller N. E.g., in a cross-sectional design, at least 34 subjects/group are needed before the BF can reach a threshold of ¼. C-D) Percentage of BF showing support (1/BF > 4) for H0 at different N. E.g., at 50 subjects/group, only 45% of BF will show support in favor of H0, when H0 is true. Hence, in order to stop for H0 when using commonly seen sample sizes in PET studies, we recommend to use a one-tailed BF t-test instead. This means that the researchers must make a prediction of the direction of the effect before initiating the study.

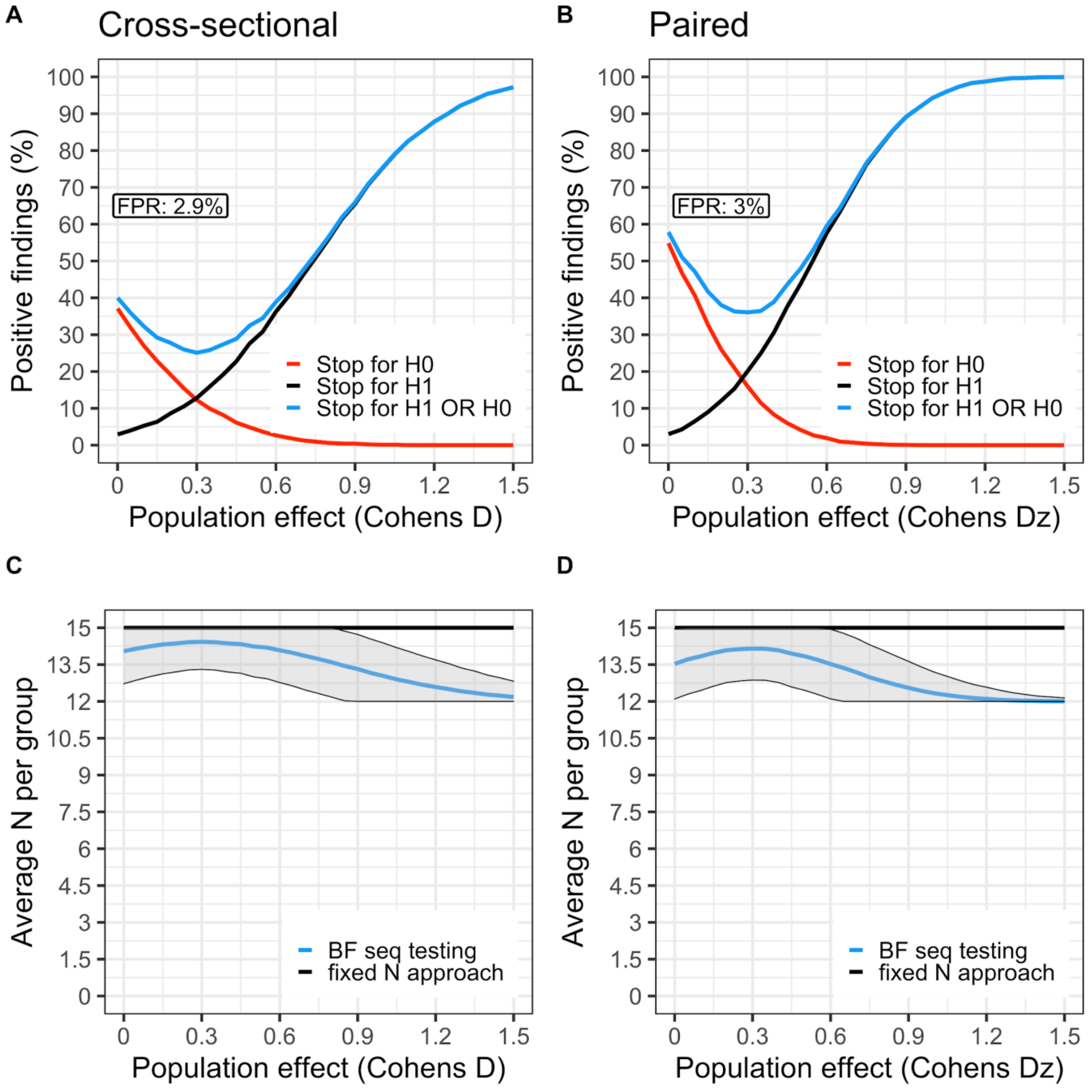

Figure S7. Settings for the simulation: H1 is described by one-sided Cauchy (0,0.707), (Nstart = 12, Nmax = 15, BF threshold = 4). A and B) The black curve shows the proportion of studies that showed support for H1 (BF>4) during data collection, at a range of population effects (starting at no effect, D = 0). The red curve is the proportion of studies showing support for H0 (BF<¼). The blue curve is the sum of the red and black curves. C and D) shows the average number of subjects needed to reach a stopping decision at different population effects. The flat black line represents Nmax (15 subjects/group). BF sequential testing is the blue line with shaded area denoting ± 1 SD.

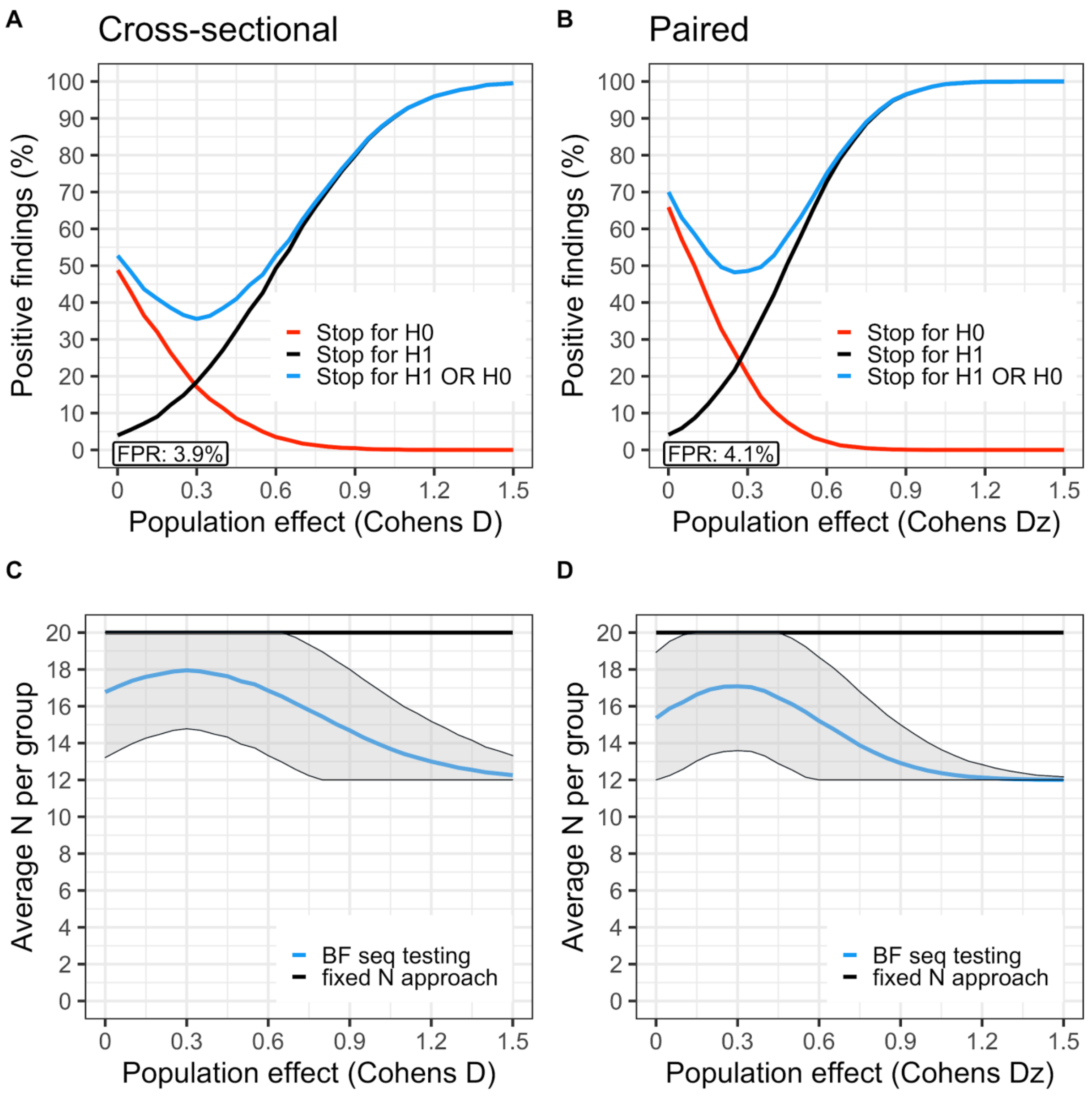

Figure S8. Settings for the simulation: H1 is described by one-sided Cauchy(0,0.707), (Nstart = 12, Nmax = 20, BF threshold = 4). A and B) The black curve shows the proportion of studies that showed support for H1 (BF>4) during data collection, at a range of population effects (starting at no effect, D = 0). The red curve is the proportion of studies showing support for H0 (BF<¼). The blue curve is the sum of the red and black curves. C and D) shows the average number of subjects needed to reach a stopping decision at different population effects. The flat black line represents Nmax (20 subjects/group). BF sequential testing is the blue line with shaded area denoting ± 1 SD.

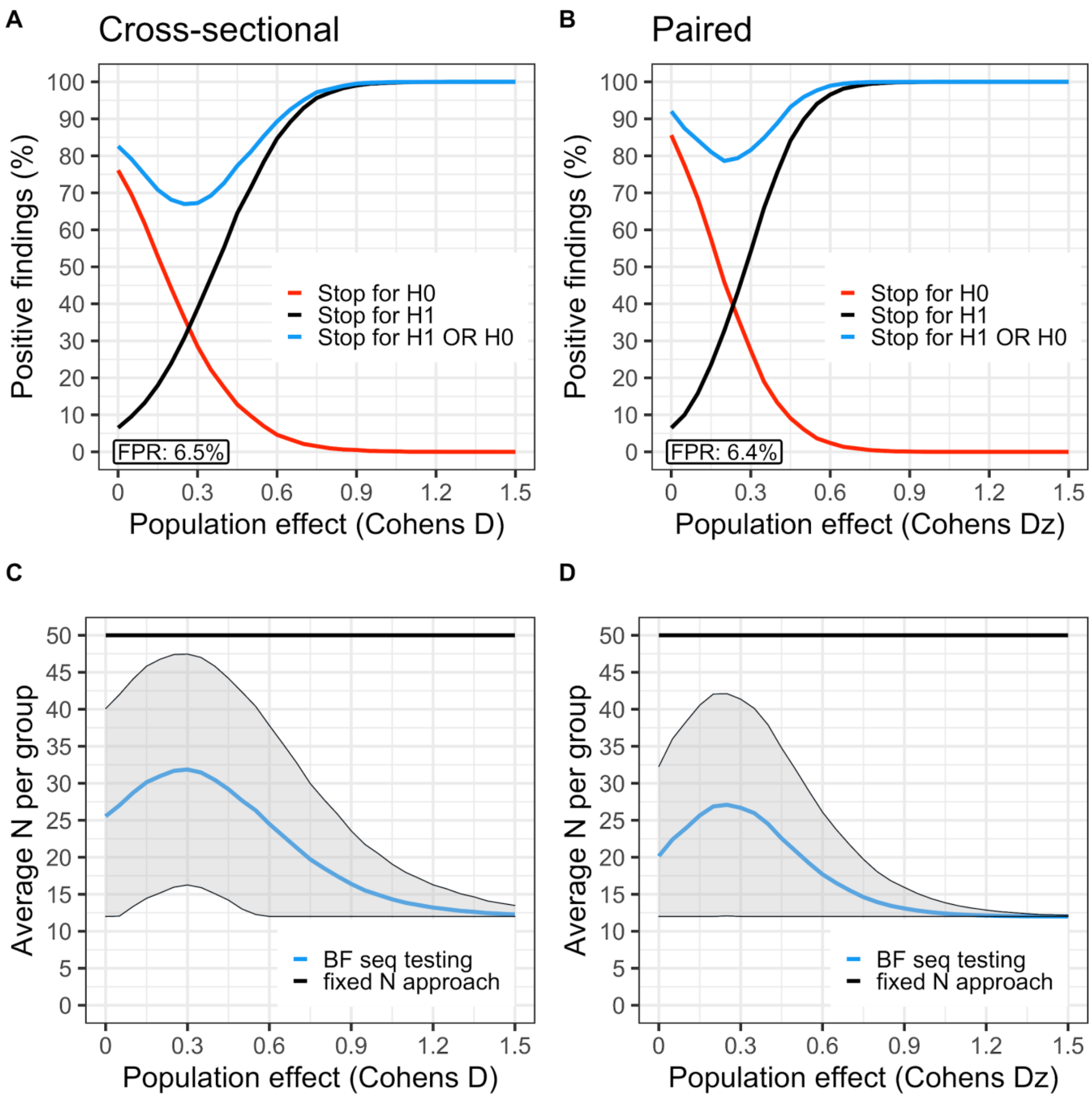

Figure S9. Settings for the simulation: H1 is described by one-sided Cauchy (0,0.707), (Nstart = 12, Nmax = 50, BF threshold = 4). A and B) The black curve shows the proportion of studies that showed support for H1 (BF>4) during data collection, at a range of population effects (starting at no effect, D = 0). The red curve is the proportion of studies showing support for H0 (BF<¼). The blue curve is the sum of the red and black curves. C and D) shows the average number of subjects needed to reach a stopping decision at different population effects. The flat black line represents Nmax (50 subjects/group). BF sequential testing is the blue line with shaded area denoting ± 1 SD.

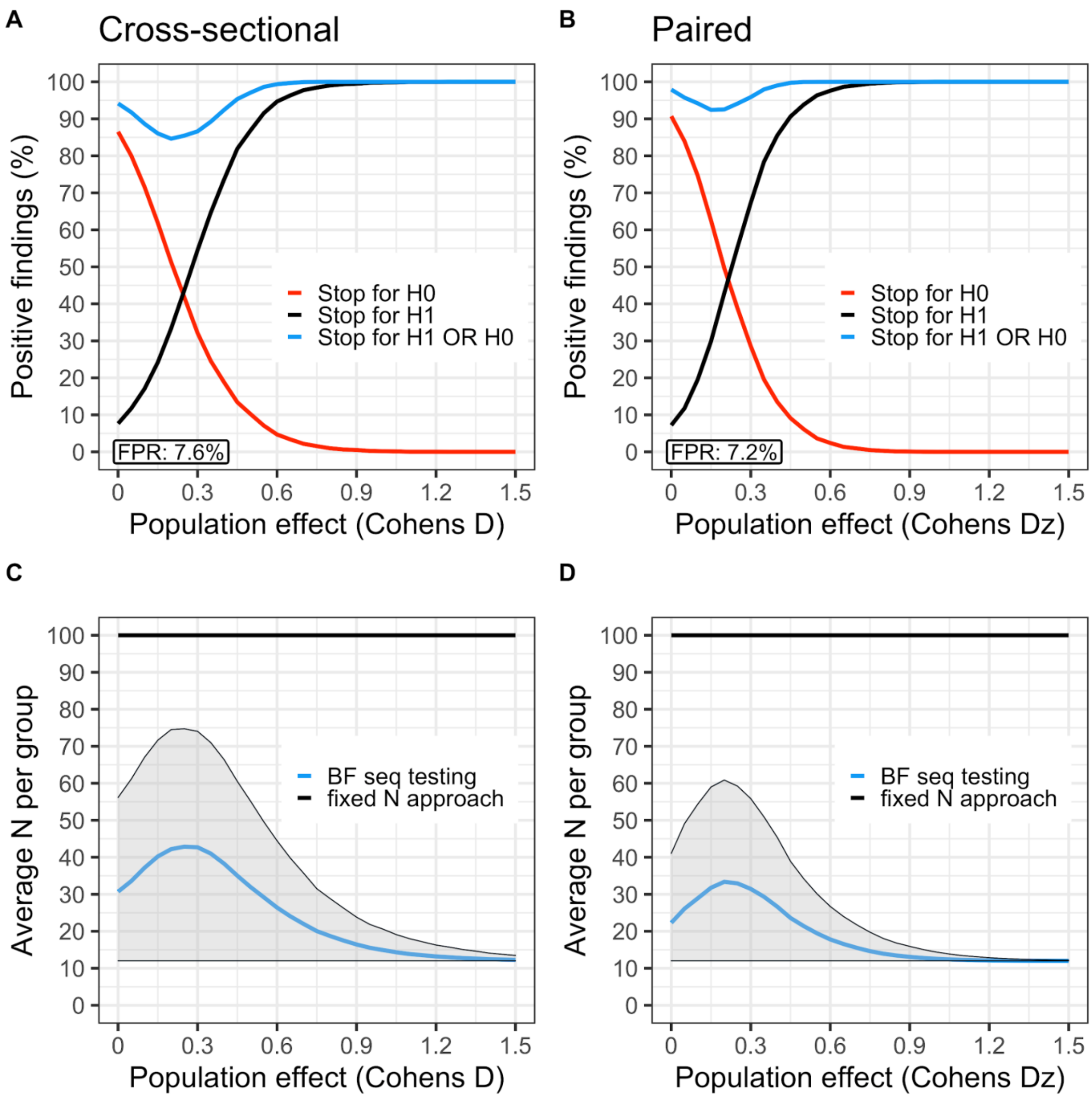

Figure S10. Settings for the simulation: H1 is described by one-sided Cauchy(0,0.707), (Nstart = 12, Nmax = 100, BF threshold = 4). A and B) The black curve shows the proportion of studies that showed support for H1 (BF>4) during data collection, at a range of population effects (starting at no effect, D = 0). The red curve is the proportion of studies showing support for H0 (BF<¼). The blue curve is the sum of the red and black curves. C and D) shows the average number of subjects needed to reach a stopping decision at different population effects. The flat black line represents Nmax (100 subjects/group). BF sequential testing is the blue line with shaded area denoting ± 1 SD.

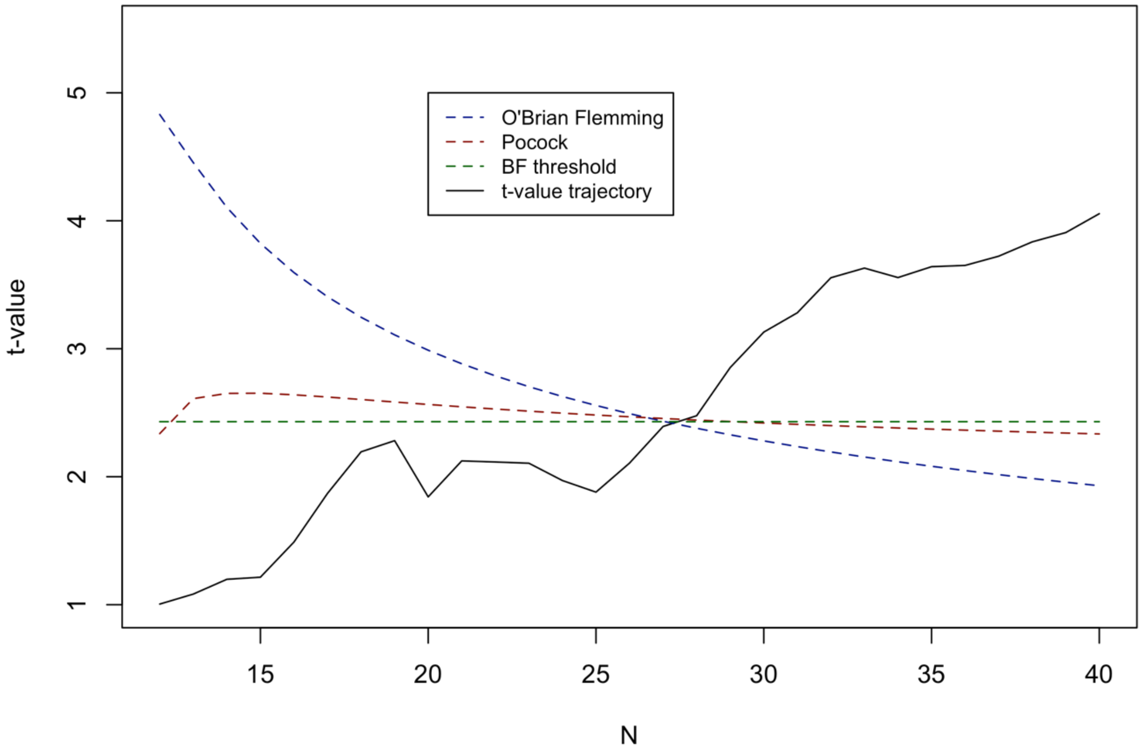

Figure S11. Comparison between sequential BF testing and two common NHST alpha spending approaches, using the real clinical data set from Objective 3 in main text, where 40 patients and major depressive disorder and 40 healthy control subjects were examined with PET [11C]WAY100635. Nstart = 12, BF threshold = 5, alpha = 0.05. A BF of 5 was selected to correspond to a false positive rate of not more than 5%. In this data set all three approaches showed similar results, with a stop decision being made at 27 subjects/group finding support for H_1 (BF) or rejecting H_0 (NHST).

The O'Brian Flemming NHST alpha spending approach starts out with a conservative stopping boundary, and gradually becomes more liberal for each intermittent test. The Pocock boundary starts out with a more liberal boundary, increases slightly and then gradually decreases towards the end of the sequential testing. Assuming there is a large population effect, the Pocock and BF approaches will, on average, be able to stop at an earlier phase of the study. However, if the population effect is of smaller size, than the O'Brian Flemming approach might be more suitable, since it has a greater average chance finding a true positive towards the end of the study. If PET researchers still whishes use sequential BF testing, but is interested in obtaining similar stopping characteristics as showed by O'Brian Flemming approach, they can plan a study that starts out with a high BF threshold and then gradually decrease it for each intermittent test. Such an approach is however outside the scope of this tutorial.

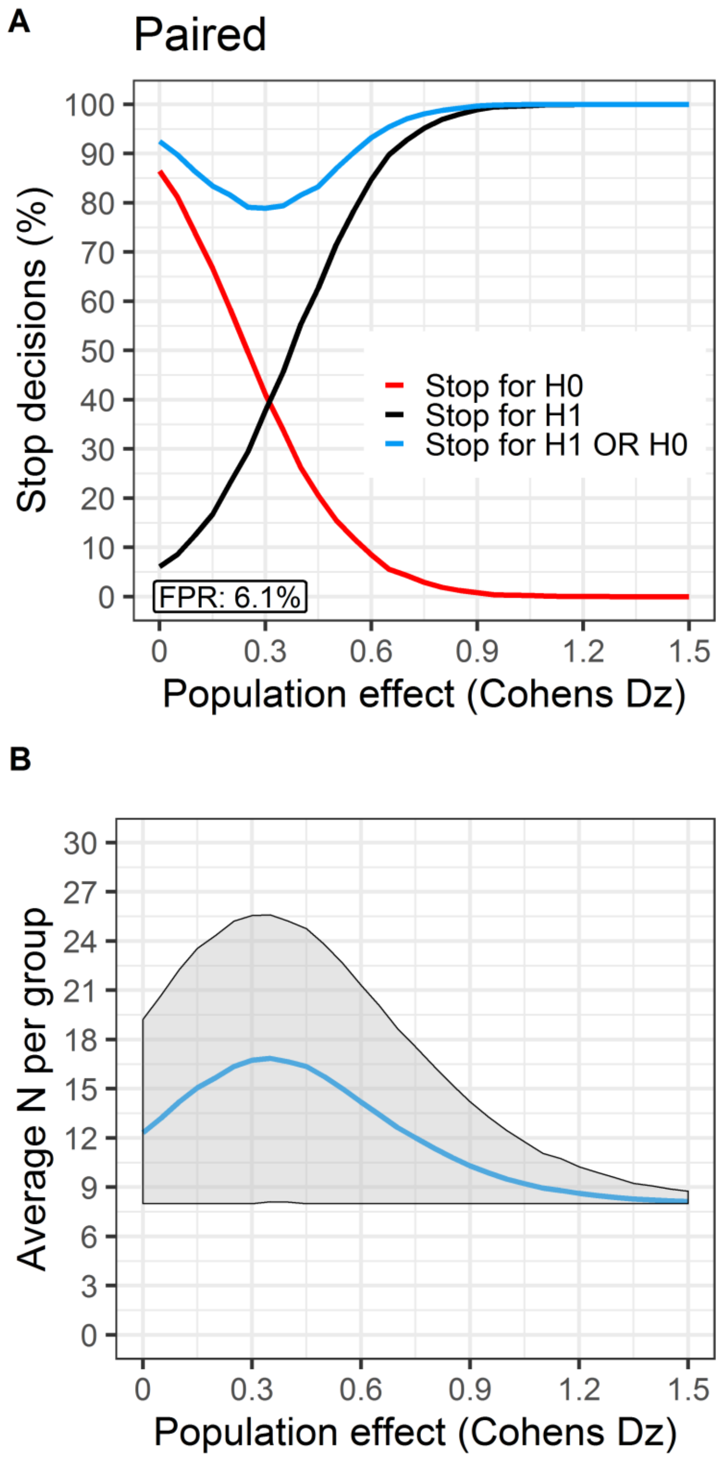

Figure S12 Settings for the simulation: H1 is a one-sided Cauchy(0,1), (Nstart = 8, Nmax = 30, BF threshold = 4). A) The black curve shows the proportion of studies that showed support for H1 (BF>4) during data collection, at a range of population effects (starting at no effect, D = 0). The red curve is the proportion of studies showing support for H0 (BF<¼). The blue curve is the sum of the red and black curves. B) shows the average number of subjects needed to reach a stopping decision at different population effects. The flat black line represents Nmax (30 subjects/group). BF sequential testing is the blue line with shaded area denoting ± 1 SD. The fact that a lower average sample size trades off against a higher risk of false negatives can be seen by comparing this figure to panel B and

D in Figure 5 from the main text which have the same settings except for: 1) $H1\colon \delta \sim Cauchy(0, 0.707)$ $instead\ of$ $H1\colon \delta \sim Cauchy(0, 1)$ *and 2) testing starts at 12 subjects.*

Increasing the width of the Cauchy can be desirable in e.g., a paired study design, where the variance can be expected to be low, but the difference in raw scores is assumed to be similar to that from a cross-sectional design. The reason for this is that the same change in raw score will correspond to a much larger standardized effect size (see Table 2 in main text), and it is therefore sensible to specify an H_1 which predicts a more extreme difference. The lower rate of false positives that results from using a higher *r* (e.g., 1 instead of 0.707) can be utilized by starting the sequential BF testing earlier than at 12 subjects/group. As a result, the average sample size needed to reach a decision can be further decreased.

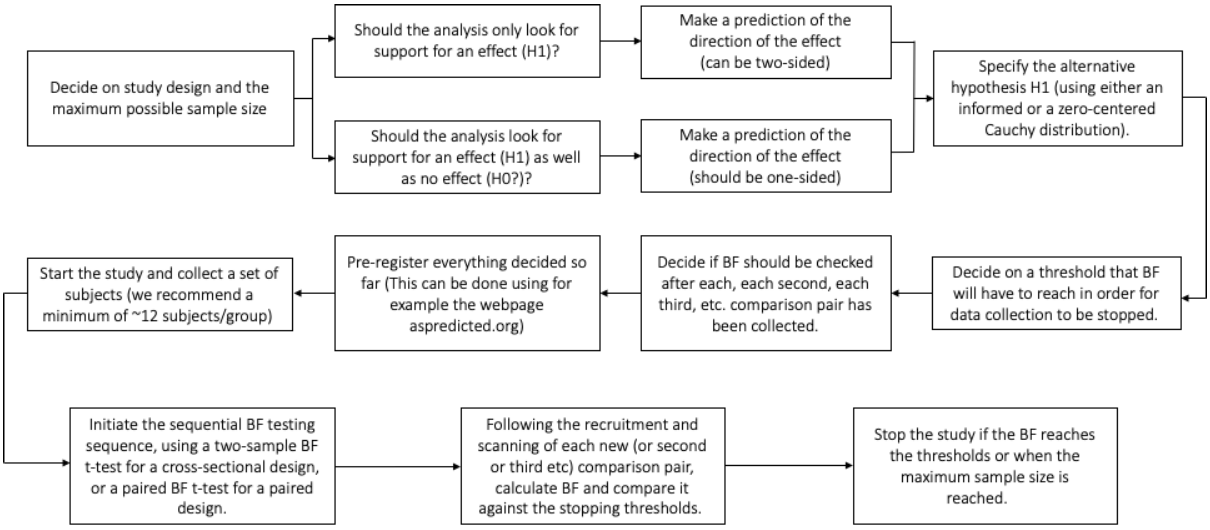

Figure S13. Recommended steps to follow in order to perform a clinical PET study using sequential BF testing, for a paired or cross-sectional design.
